# Task-state skin potential abnormalities can distinguish major depressive disorder and bipolar depression from healthy controls

**DOI:** 10.1038/s41398-024-02828-9

**Published:** 2024-02-23

**Authors:** Hailong Lyu, Huimin Huang, Jiadong He, Sheng Zhu, Wanchu Hong, Jianbo Lai, Tongsheng Gao, Jiamin Shao, Jianfeng Zhu, Yubo Li, Shaohua Hu

**Affiliations:** 1 Department of Psychiatry, The First Affiliated Hospital, Zhejiang University School of Medicine; Key Laboratory of Mental Disorder’s Management of Zhejiang Province, Hangzhou, 310003 China; 2 Brain Research Institute of Zhejiang University, Hangzhou, 310003 China; 3 Zhejiang Engineering Center for Mathematical Mental Health, Hangzhou, 310003 China; 4 The Third Affiliated Hospital of Wenzhou Medical University, Wenzhou, 325200 China; 5 Ruian People’s Hospital, Wenzhou, 325200 China; 6 College of Information Science and Electronic Engineering, Zhejiang University, Hangzhou, 310027 China; 7 Department of Psychiatry, The Ruian Fifth People’s Hospital, Wenzhou, 325200 China; 8 Ningbo Psychiatric Hospital, Ningbo, 315032 China

**Keywords:** Bipolar disorder, Depression, Diagnostic markers, Physiology

## Abstract

Early detection of bipolar depression (BPD) and major depressive disorder (MDD) has been challenging due to the lack of reliable and easily measurable biological markers. This study aimed to investigate the accuracy of discriminating patients with mood disorders from healthy controls based on task state skin potential characteristics and their correlation with individual indicators of oxidative stress. A total of 77 patients with BPD, 53 patients with MDD, and 79 healthy controls were recruited. A custom-made device, previously shown to be sufficiently accurate, was used to collect skin potential data during six emotion-inducing tasks involving video, pictorial, or textual stimuli. Blood indicators reflecting individual levels of oxidative stress were collected. A discriminant model based on the support vector machine (SVM) algorithm was constructed for discriminant analysis. MDD and BPD patients were found to have abnormal skin potential characteristics on most tasks. The accuracy of the SVM model built with SP features to discriminate MDD patients from healthy controls was 78% (sensitivity 78%, specificity 82%). The SVM model gave an accuracy of 59% (sensitivity 59%, specificity 79%) in classifying BPD patients, MDD patients, and healthy controls into three groups. Significant correlations were also found between oxidative stress indicators in the blood of patients and certain SP features. Patients with depression and bipolar depression have abnormalities in task-state skin potential that partially reflect the pathological mechanism of the illness, and the abnormalities are potential biological markers of affective disorders.

## Introduction

Major depressive disorder (MDD) and bipolar disorder (BD) are two prevalent mood disorders characterized by significant abnormalities in mood and emotional states [1]. However, the early identification of these disorders remains a challenging due to the lack of valuable biological markers. Many individuals with MDD are not diagnosed early, with only 47.3% being identified by general practitioners [2, 3]. Similarly, BD often presents as a depressive episode, leading to misdiagnosis in 25% of BD patients, with a substantial delay of 5-10 years before receiving the correct diagnosis [2, 4]. Extensive research has been conducted in various fields, including neuroimaging, biochemistry, genetics, and epigenetics, to identify biological markers for mood disorders [5–8]. However, the practical application of these markers is limited by factors such as high cost, poor feasibility, or inadequate specificity. In light of these challenges, electrodermal activity has emerged as a potential biomarker for mood disorders due to its non-invasiveness, ease of monitoring, affordability, and high sensitivity to the emotional perception [9–11].

Electrodermal activity is a sensitive physiological indicator of changes in sympathetic nervous system activity, reflecting the electrical phenomenon occurring in the sweat glands, dermis, and epidermis tissues innervated by the sympathetic nervous system [12, 13]. It provides valuable insights into physiological arousal and, due to its subconscious nature, offers and objective means to investigate emotions [14]. Skin conductance detection and skin potential (SP) detection are the methods employed to measure electrodermal activity. The former measures the changes in electrical resistance by applying a small external current to two skin-attached electrodes, while the latter directly measures the natural potential difference of the limb skin without any external current. Previous studies have demonstrated that patients with MDD exhibit a diminishing performance of skin conductance in response to repeated non-significant stimulus [15]. Another study found that incorporating skin conductance differences between resting and task states into a decision tree classification model achieved a 74% accuracy in distinguishing depression patients from healthy controls [16]. However, the measurement of skin conductance may cause discomfort due to the additional electrical stimulation, and its results may be influenced by physiological factors such as gender and age [17, 18].

On the other hand, SP has been suggested to provide unique psychological information. However, detecting SP signals can be challenging and complex due to variations in both positive and negative directions and the existence of monophasic, biphasic, and triphasic responses [19–22]. In this study, we have developed a wearable wireless device to measure SP signals between the middle finger and the left wrist. Preliminary experiments have demonstrated the accurate identification of four distinct emotional states (happiness, sadness, anger, fear), elicited by video stimuli using participants’ SP signals alone, achieving a 75% accuracy [23]. These findings suggest that SP signals hold promise as a viable approach for the objective assessment of emotions. Recent hypotheses propose that the SP difference may be attributed to a high concentration of superoxide radicals in the connective tissue, and elevated levels of oxidants, including superoxide free radicals and nitric oxide, have been implicated in oxidative stress and the pathogenesis/physiology of mood disorders [24–26]. Therefore, exploring the application of SP as a potential biomarker for the diagnosis of mood disorders is warranted.

**Fig. 1:**
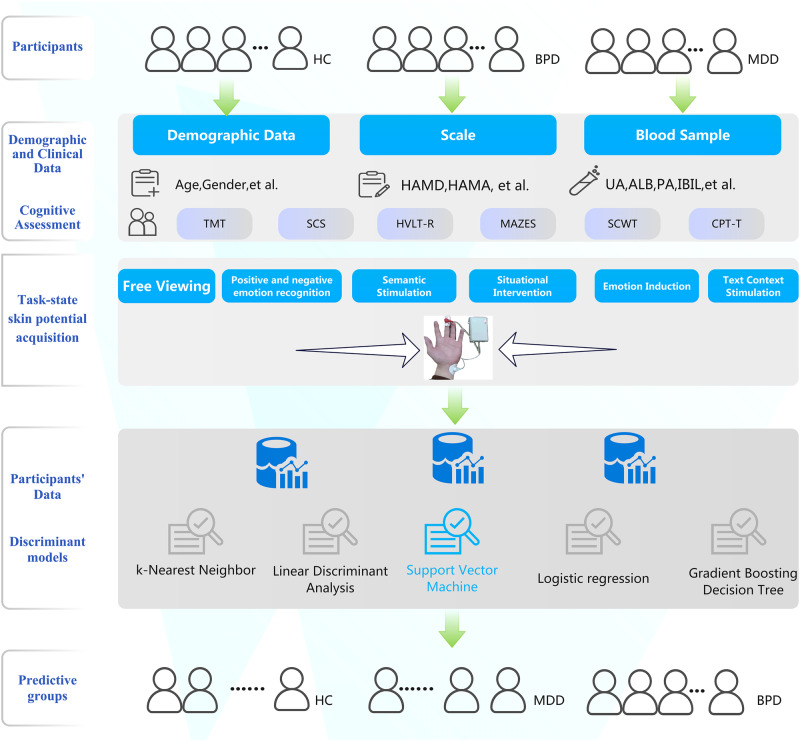
Research steps to distinguish patients with mood disorders from healthy controls using machine-learning discriminant models based on task-state skin potential data. In the present study, hematological samples of healthy controls were not collected. A total of five discriminant models were constructed, and the SVM model differentiated groups with the highest classification accuracy. The accuracy of this model in distinguishing major depressive disorder from healthy controls was 78%, and the accuracy in correctly classifying participants into three groups was 59%. BPD, bipolar depressive disorder; MDD, major depressive disorder (unipolar depression); CON, healthy controls.

To date, few studies have utilized machine learning models to differentiate patients with mood disorders from healthy controls using SP signals during task states. In this study, we recruited patients with major depressive disorder (MDD), bipolar depression (BPD), and healthy controls, and measured SP signals during various emotion-inducing tasks using a customized wearable device. Multiple discriminant models were constructed to distinguish patients from healthy controls by the difference in SP signals. Additionally, we explored the correlation between participants’ SP signals and blood indicators of oxidative stress (Fig. 1).

## Methods

### Participant

Seventy-seven patients with BPD and 53 patients with MDD were recruited in the First Affiliated Hospital of Zhejiang University School of Medicine, Zhejiang, China. A total of 79 healthy controls were recruited through word-of-mouth or recruitment advertisements at Zhejiang University and the local community. All participants were between the ages of 15 and 55 and had received at least a middle school education. Diagnosis of the disease was based on the Diagnostic and Statistical Manual of Mental Disorder (DSM-IV) criteria. The researchers conducted the Mini International Neuropsychiatric Interview (MINI) screening for all patients to confirm their diagnosis. Healthy participants were asked to confirm that there were no first-degree relatives with schizophrenia, bipolar disorder, or depression. Exclusion criteria for the patient group included: 1) meeting diagnostic criteria for schizophrenia and related spectrum disorders; 2) Young Mania Rating Scale score greater than 6; 3) history of severe head injury (loss of consciousness for more than 5 minutes), current or past epilepsy, intracranial hypertension, or other severe neurological disorders; 4) history of alcohol or substance abuse/dependence within the 6 months prior to testing.

The following scales were used to assess the severity of participants’ anxiety and depressive mood states, including the Beck Depression Inventory (BDI-II), the Beck Anxiety Inventory (BAI), the 17-item version of the Hamilton Depression Rating Scale (HAMD) and the Hamilton Anxiety Rating Scale (HAMA). Blood samples from the participants were collected to test the following indicators: cortisol levels, adrenocorticotropic hormone, uric acid, indirect bilirubin levels, direct bilirubin levels, and prealbumin. All participants were tested for cognitive function, and the assessment instruments included the Trail Making Test, the Symbol Coding Test, the Hopkins Verbal Learning Test-Revised (HVLT-R), the Neuropsychological Assessment Battery-mazes subtest (MAZES), the Stroop Color and Word Test, and the Continuous Performance Test-Identical Pair (CPT-IP). Please see Table S2 in the supplementary material for a detailed description of the cognitive functioning tests.

Ethical approval was granted by the Hospital Ethical Committee in The First Affiliated Hospital of Zhejiang University School of Medicine. All participants or their guardians agreed to participate in this study and signed a written informed consent form.

### Apparatus and stimuli

The collection of skin potential data was completed using a customized professional device (model: CTP008) consisting of a small box that contains electronic components and two electrodes. The two electrodes were attached to the middle finger and left wrist of all participants. Please refer to our previous paper for more information about the signal acquisition principle of this device and its details [23]. The device collects SP signals at a sampling rate of 5 Hz. The data was transmitted via Bluetooth to the Mood Monitor Assistant app installed on a smartphone. The collection of skin potential data is synchronized with the presentation time of the stimuli. All participants were instructed to view six stimulus tasks in sequence, which consisted of pictures, videos, or text with specific emotional content. For a detailed description of these six tasks, please refer to Table S1 in the supplementary materials. They are: 1) free viewing task, 2) positive and negative emotion recognition task, 3) semantic stimulation task, 4) situational intervention task, 5) emotion induction task, and 6) text context stimulation task. Stimuli were displayed on a 24-inch monitor with a refresh rate of 60 Hz and were presented in the center of the screen. All participants viewed stimuli at 100 cm. The eyes are aligned with the upper third of the display screen. A camera was placed on the monitor to record the participants’ facial expression information. The researchers used the video data to confirm that the participants were viewing the content on the monitor during the experiment. (Figure S1)

### Feature extraction

The data extraction and pre-processing procedures were conducted using Python 3.1. These procedures involved outlier removal, interpolation, and normalization. Each sample data was normalized separately based on specific tasks. Furthermore, to enhance the quality of the SP data, denoising techniques were applied. This involved utilizing a low-pass filtering module in the hardware as well as employing wavelet transform. Wavelet transform is a commonly used method in bio-signal analysis studies [23, 27].

In our study, a total of 18 feature values were derived for each task, including 10 time-domain features and 8 time-frequency domain features. Table 1 provides a detailed overview of these feature values. For the time-domain features, all characteristics, except for the maximum and minimum values of raw voltage, were computed after normalizing the raw SP signal. The computation of time-frequency domain feature energy values was performed using the PyWavelets library in Python. Wavelet packet decomposition, which encompasses dual time-frequency analysis, multi-resolution analysis, and arbitrary multi-scale transformation, which utilized to facilitate the time-frequency analysis of SP signals.

**Table 1:** Measures and definition of skin potential characteristics.

characteristics	Measures	Definition
*Time domain*		
	max	Maximum value of the original voltage before normalization (in mV)
	min	Minimum value of the original voltage before normalization (in mV)
	n50	Median
	mean	Mean value
	var	Variance
	rms	Root mean square
	diff1.mean	Mean value of first-order differentiation
	diff2.mean	Mean of second-order differentiation
	diff1.std	Standard deviation of first-order differentiation
	diff2.std	Standard deviation of second-order differentiation
*Time-frequency domain*		
	freq0	0–0.0625 Hz band energy
	freq1	0.0625–0.125 Hz band energy
	freq2	0.125–0.1875Hz band energy
	freq3	0.1875–0.25 Hz band energy
	freq4	0.25–0.3125 Hz band energy
	freq5	0.3125–0.375 Hz band energy
	freq6	0.375–0.4375 Hz band energy
	freq7	0.4375–0.5 Hz band energy

In the time domain characteristics, except for max and min, all the measures were calculated by normalizing the original skin potential signal. The energy calculation of the time-frequency domain measures is mainly performed by wavelet packet decomposition.

### Data analysis

Basic statistical analysis was performed in R 4.2.2 (https://www.R-project.org/). One-way analysis of variance (ANOVA) was employed to analyze quantitative data, such as age and SP features. Post hoc multiple comparisons were performed using Bonferroni or Tamhane’s T2 correction for inter-group comparisons. Independent two-sample t-tests were utilized to compare two sets of quantitative data, such as HAMD scores. Chi-square tests or rank-sum tests were employed to compare qualitative data, including gender, education level, and clinical characteristics. Statistical significance was defined as a two-tailed p-value less than 0.05.

For discriminant analysis and model performance comparison, the scikit-learn machine learning library in Python was utilized. Several discriminant models, including K-Nearest Neighbor, Linear Discriminant Analysis, Support Vector Machine (SVM), Logistic Regression, and Gradient Boosting Decision Tree, were evaluated for their accuracy in distinguishing patients form healthy controls using SP data. The reported discriminant results in this study were obtained using the SVM model.

SVM is a machine learning algorithm that finds the optimal hyperplane to separate data points into different classes. It creates a decision boundary with the maximum possible margin from the sample points, thereby improving the generalization performance. The hyperparameter settings of the SVM model we set as follows: a penalty coefficient of 1.3, a kernel function parameter of 0.0085, and the Radial Basis Function as the choice of kernel function. To circumvent class imbalance, 50 participants were randomly selected from each group of BPD patients, depression patients, and healthy controls for inclusion in the discriminant model. Leave-one-out cross-validation was used to evaluate the performance of the machine learning model. Sensitivity, specificity, accuracy, F1 score, and area under the ROC were employed as performance metrics for the model evaluation.

## Results

### Demographic and clinical characteristics

A total of 77 patients with bipolar depression (BPD), 53 patients with major depressive disorder (MDD), and 79 healthy controls participated in this study. Detailed demographic and clinical characteristics are summarized in Table 2. The mean age of BPD patients was significantly lower than the other two groups (*P* < 0.01). Healthy controls had a significantly higher level of education compared to BPD and MDD patients (*P* < 0.01). There was no significant statistical difference in the Hamilton Depression Rating Scale (HAMD) and Hamilton Anxiety Rating Scale (HAMA) scores between BPD and MDD patients. Both BPD and MDD patients performed significantly worse than healthy controls in all six cognitive tests (*P* < 0.05), but there was no statistical difference between the two patient groups (*P* > 0.05).

**Table 2:** Demographic and clinical characteristics of patients with bipolar depressive disorder, major depressive disorder, and healthy controls.

	BPD (*n* = 77) Mean ± SD	MDD (*n* = 53) Mean ± SD	HC (*n* = 79) Mean ± SD	t/F	*P* value	Corrected *P* value		
						BPDvsHC	MDDvsHC	BPDvsMDD
Sex, M/F	26/51	17/36	27/52	0.067^a^	0.967			
Age (year)	18.92 ± 3.88	22.72 ± 8.49	25.3 ± 4.86	25.025	<0.001	<0.001	0.123	0.01
Education	2(1, 2.5)	2(2, 3)	3(3, 3)	105.689^a^	<0.001	<0.05	<0.05	/
HAMD	18.13 ± 5.76	16.68 ± 5.99	/	1.387	0.168	/	/	/
HAMA	18.08 ± 9.10	17.15 ± 9.17	/	0.568	0.571	/	/	/
BDI-II	32.73 ± 14.55	30.19 ± 11.32	/	164.152	<0.001	<0.001	<0.001	0.605
BAI	42.87 ± 12.70	40.38 ± 13.75	/	76.948	<0.001	<0.001	<0.001	0.653
*Cognitive function performance*								
Trail Making Test	42.15 ± 16.09	44.69 ± 16.54	27.01 ± 7.95	34.615	<0.001	<0.001	<0.001	0.908
Symbol Coding Test	53.36 ± 11.32	54.53 ± 10.00	69.05 ± 9.39	53.881	<0.001	<0.001	<0.001	1.000
HVLT-R	23.94 ± 5.14	24.64 ± 5.13	28.44 ± 4.30	19.057	<0.001	<0.001	<0.001	1.000
MAZES	18.45 ± 5.74	18.87 ± 4.97	21.57 ± 3.87	8.973	<0.001	<0.001	0.007	1.000
SCWT	105.52 ± 38.50	102.00 ± 29.81	79.93 ± 24.37	14.561	<0.001	<0.001	<0.001	1.000
CPT-IP	2.15 ± 0.75	2.33 ± 0.81	3.00 ± 0.04	35.199	<0.001	<0.001	<0.001	0.381
*Blood test indicators*								
Cortisol (ug/dl)	10.41 ± 3.61	11.1 ± 4.43						
ACTH (ph/ml)	21.47 ± 12.42	22.59 ± 10.72						
Uric Acid (umol/l)	335.74 ± 95.60	319.79 ± 89.70						
IBIL (umol/l)	5.15 ± 4.35	5.78 ± 4.56						
DBIL (umol/l)	3.61 ± 1.91	3.92 ± 2.13						
Albumin (umol/l)	44.36 ± 3.14	44.03 ± 3.09						
Prealbumin (mg/dl)	26.43 ± 5.89	24.34 ± 5.40						

*BPD* bipolar depression, *MDD* major depressive disorder, *HC* healthy controls, *HAMD* Hamilton depression rating scale, *AMA* Hamilton anxiety rating scale, *BDI-II* Beck depression inventory second version, *AI* Beck anxiety inventory, *HVLT-R* Hopkins verbal learning test—revised; *MAZES* neuropsychological assessment attery, mazes subtest, *SCWT* The Stroop color and word test, *CPT-IP* continuous performance test-identical pair, *ACTH* adrenocorticotropic hormone, *IBIL* indirect bilirubin levels, *DBIL* direct bilirubin levels, Education was coded as follows: 1 = primary high school, 2 = junior high school, 3 = senior high school, 4 = Bachelor’s degree or above. Shown in the table as median and upper and lower quartiles. The corrected p-values were analyzed using the Bonferroni method.

a: Pearson’s Chi-square test.

The mean duration of BPD in this study was 3.9 years. At the start of the study, 2 BPD patients did not use any psychiatric-related medications, 32 patients used only 1 mood stabilizer, 34 patients used 2 mood stabilizers, and 9 patients used more than 2 mood stabilizers. The mean duration of MDD in the patients in this study was 3.7 years. Until the start of the study, 1 depressed patient did not use any psychiatric-related medication, 46 patients used only 1 antidepressant, and 6 patients used 2 or more antidepressants.

### Comparison of SP characteristics under task conditions

The comparison results of SP characteristics in BPD, MDD, and healthy controls under different task conditions are summarized in Table 3 and Table S3-S8 (supplementary materials). No significant differences in SP characteristics were found among the three groups during the task of viewing textual stimuli. However, SP abnormalities were detected in MDD patients compared to healthy controls in all other tasks. Task-related SP abnormalities were also observed in BPD patients during the free viewing and emotion induction tasks. No significant differences in task-related SP were found between MDD and BPD patients in pairwise comparisons among the three groups.

**Table 3:** The task state skin potential characteristics with significant differences between three groups comparing patients with BPD, MDD, and healthy controls.

	BPD (*n* = 77)	MDD (*n* = 53)	HC (*n* = 79)	F value	*P* value	Corrected *P* value		
						BPDvsHC	MDDvsHC	BPDvsMDD
task1.diff2_std	0.021 ± 0.015	0.019 ± 0.014	-0.015 ± 0.010	3.947	0.021	0.018	0.189	0.905
task1.freq2	0.081 ± 0.057	0.105 ± 0.075	0.074 ± 0.043	4.408	0.013	0.854	0.034	0.158
task1.freq4	0.049 ± 0.037	0.065 ± 0.045	0.047 ± 0.031	4.093	0.018	0.985	0.046	0.105
task1.freq5	0.031 ± 0.023	0.042 ± 0.032	0.030 ± 0.019	4.126	0.017	0.976	0.059	0.131
task2.var	0.060 ± 0.022	0.059 ± 0.022	0.068 ± 0.0262	3.118	0.046	0.100	0.111	1.000
task2.freq3	0.080 ± 0.047	0.097 ± 0.054	0.071 ± 0.041	5.114	0.007	0.624	0.005	0.127
task2.freq4	0.066 ± 0.041	0.081 ± 0.048	0.059 ± 0.039	4.137	0.017	0.917	0.014	0.166
task2.freq5	0.046 ± 0.032	0.052 ± 0.043	0.038 ± 0.024	3.076	0.048	0.395	0.047	0.868
task3.diff1_std	0.029 ± 0.012	0.035 ± 0.0171	0.028 ± 0.011	4.398	0.013	0.965	0.047	0.111
task3.diff2_std	0.025 ± 0.015	0.033 ± 0.030	0.021 ± 0.013	6.534	0.002	0.248	0.019	0.169
task3.freq7	0.036 ± 0.025	0.044 ± 0.023	0.034 ± 0.016	3.326	0.038	0.890	0.030	0.224
task4.diff2_mean	-5.577×10^-6^ ± 8.068×10^-5^	-2.185×10^-5^ ± 9.289×10^-5^	1.559×10^-5^ ± 6.055×10^-5^	3.879	0.022	0.266	0.021	0.718
task4.freq1	0.149 ± 0.087	0.158 ± 0.085	0.123 ± 0.081	3.144	0.045	0.182	0.064	1.000
task4.freq3	0.074 ± 0.050	0.086 ± 0.054	0.063 ± 0.044	3.343	0.037	0.533	0.032	0.537
task5.n50	0.435 ± 0.150	0.418 ± 0.130	0.486 ± 0.140	4.292	0.015	0.078	0.023	1.000
task5.mean	0.447 ± 0.115	0.429 ± 0.103	0.485 ± 0.106	4.691	0.010	0.095	0.012	1.000
task5.rms	0.499 ± 0.108	0.477 ± 0.102	0.530 ± 0.107	4.181	0.017	0.208	0.016	0.734
task5.freq0	2.513 ± 0.616	2.450 ± 0.560	2.773 ± 0.618	5.660	0.004	0.023	0.009	1.000

Note. Please see Table 1 for the definition of the skin potential measures. This table lists only those measures that showed statistically significant differences when compared between groups. Please see the supplemental file for a summary of results for the remaining measures. The names of the tasks referred to by each number are as follow. Task 1, free-viewing task; task 2, positive-negative emotion recognition task; task 3, semantic stimulus task; task 4, situational intervention task; task 5, emotional induction task. *BPD* bipolar depression, *MDD* major depressive disorder, *HC* healthy control.

### Predictive performance of the discriminant model

**Fig. 2:**
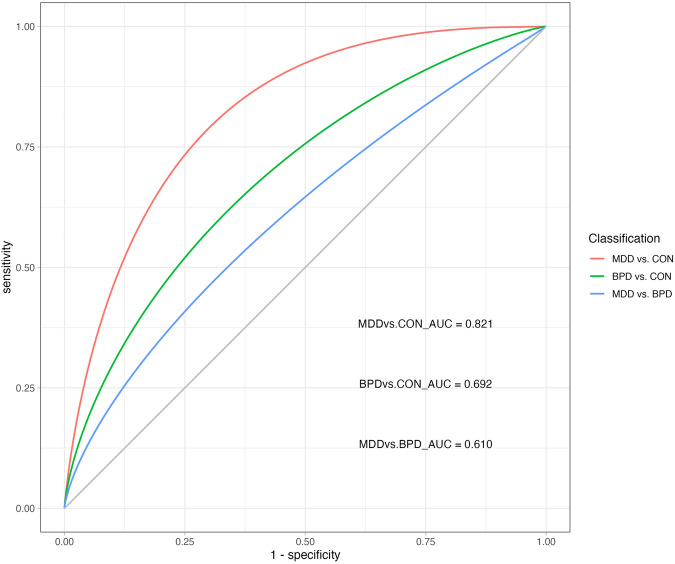
ROC curves of the support vector machine model discriminating bipolar depression, unipolar depression, and healthy controls based on skin potential characteristics. ROC receiver operating characteristic, AUC area under the curve, BPD bipolar depressive disorder, MDD major depressive disorder (unipolar depression); CON healthy controls.

Fifty participants were randomly selected from each of the three groups (BPD, MDD, and healthy controls), and their SP data were used to construct disease discriminant models for separating patients from healthy controls. Five different models were developed to assess their discriminatory validity. In this study, a total of 18 SP features were detected for each task, and all 108 indicators for the 6 tasks were included in the discriminant model. The discriminant results of the five models are summarized in Table S9. Given support vector machine (SVM) model outperformed other models, the results of SVM will be primarily reported. (Table 4, Fig. 2)

**Table 4:** The confusion matrix of the support vector machine model for discriminant analysis of patients with BPD, MDD, and healthy controls based on skin potential characteristics.

		Predicted Class			Sensitivity	Specificity	Accuracy	Precision	F1 score	AUC
		BPD	MDD	HC						
True Class_1	MDD		37	13	0.74	0.82	0.78	0.8	0.77	0.821
	HC		9	41						
True Class_2	BPD	31		19	0.62	0.68	0.65	0.65	0.64	0.692
	HC	16		34						
True Class_3	BPD	38	12		0.76	0.62	0.69	0.67	0.71	0.61
	MDD	19	31							
True Class_4	BPD	24	9	17	0.59	0.79	0.59	0.59	0.58	/
	MDD	12	31	7						
	HC	13	4	33						

*BPD* bipolar depression, *MDD* major depressive disorder, *HC* healthy control, *AUC* area under the ROC Curve.

The SVM model built with SP features achieved an accuracy of 78% (sensitivity 78%, specificity 82%) in distinguishing MDD patients from healthy controls. It achieved an accuracy of 65% (sensitivity 62%, specificity 62%) in classifying BPD patients and healthy controls. Moreover, the SVM model demonstrated a 69% accuracy (sensitivity 76%, specificity 62%) in differentiating MDD patients from BPD patients. When classifying BPD patients, MDD patients, and healthy controls into three groups, the SVM model achieved an accuracy of 59% (sensitivity 59%, specificity 79%). (Table 4, Fig. 2).

### Correlation analysis

The correlation analysis results between SP features under task conditions and clinical features, cognitive performance, and hematological indicators are summarized in Figure S2 and Figure S3 in the supplementary material. Significant correlations were found between several SP features under different tasks and symptom scale scores of BPD and MDD patients. No significant correlations were detected between cognitive performance and SP features in either BPD or MDD patients. In BPD patients, significant positive correlations were observed between their prealbumin levels and SP time-frequency indicators under most tasks, included positive and negative emotion recognition task, semantic stimulation task, emotion induction task, and text context stimulation task (*P* < 0.05).

Furthermore, in MDD patients, significant positive correlations were found between hematological oxidative stress measures and skin conductance time-frequency indicators under tasks such as free viewing task, positive and negative emotion recognition task, emotion induction task, and text context stimulation task (*P* < 0.05).

## Discussion

In this study, we investigated skin conductance abnormalities in patients with major depressive disorder (MDD) and bipolar depression (BPD) during emotion-inducing tasks and explored the feasibility of differentiating patients from healthy controls based on these abnormalities. We designed six different video, pictorial, and textual emotion-inducing tasks participants to view, and we used a self-designed device to collect SP data. A total of 18 analysis measures were extracted from the time and time-frequency domains for each task. Our findings revealed that an SVM discriminant model based on SP measures could differentiate MDD patients from healthy controls with a 79% accuracy rate. Furthermore, the model achieved a 59% accuracy rate in distinguishing among three groups of MDD patients, BPD patients, and healthy controls. Additionally, we found significant correlations between oxidative stress indicators in the blood of patients and certain SP features.

Previous research has indicated that mood disorders are associated with abnormal processing of emotional experiences triggered by real-life stimuli. Patients with MDD tend to be overly concerned with negative emotions and show reduced sensitivity to positive emotions, while Patients with bipolar disorder may exhibit difficulties regulating emotional response when confronted with emotional stimuli [28, 29]. Emotion regulation involves the control of the limbic system by the individual’s cerebral cortex. Studies have found no difference in attention maintenance between MDD and BPD patients when viewing positive and negative facial expressions, but MDD patients showed increased activity in the dorsal anterior cingulate gyrus when viewing neutral facial expressions [30]. These findings suggest that the physiological mechanisms underlying the processing of different emotional stimuli may vary among mood disorders. Considering that SP may serve as an objective biological indicator of emotional information, we designed six different emotion-inducing tasks to investigate SP characteristics in MDD, BPD patients during the task states.

Our previous studies have demonstrated the successful acquisition of SP signals using our self-developed device [23]. In selecting of skin potential indicators, we calculated both time domain indicators and time-frequency domain measures (frequency range 0-0.0625 Hz), providing a comprehensive characterization of SP features. This approach differs from traditional analysis methods used in skin electrical activity, which typically employ Fourier transform, wavelet basis, or autoregression on raw data to obtain bioelectric signal characteristics [31]. In this study, we applied wavelet transform to obtain time-frequency domain data, as this algorithm offers good denoising capabilities and provides a stable characterization of the SP signals [27]. Our discriminant analysis demonstrated the value of these SP indicators in distinguishing patients from healthy controls, providing valuable parameters for subsequent SP studies.

Prior research suggests that a machine-learning approach to disease identification based solely on brain data should achieve an accuracy rate exceeding 75% [32]. Our study found that an SVM classifier based on SP data achieved a 79% accuracy rate in differentiating MDD patients from healthy controls. This finding is notable for two reasons. First, it highlights that electrodermal activity abnormalities are core pathological features in the affective disorders [15, 16, 33]. Second, the SVM model we constructed proved to be suitable for discriminant analysis using this type of data, as the other models we employed in this study did not achieve the desired accuracy rates. It is worth mentioning that the accuracy obtain by Kim, et al. [16], who used a decision tree classifier to separate MDD patients from healthy controls based on skin conductance data, was only 74% [16]. Another significant finding of our study was that the SVM classifier achieved a 58% accuracy rate in discriminating between bipolar depression, MDD, and healthy controls. Although this accuracy rate is nearly double that of random discrimination among the three groups (33%), it falls short of the ideal accuracy level. The overlapping pathological mechanisms between depression and bipolar disorder, as well as the fact that some first episodes of bipolar disorder present as depressive episodes pose, challenges for distinguishing between the two in both clinical practice and scientific research [1]. The average age recruited MMD participants in our study was 23 years, and it is possible that some patients may develop a manic episode in the future. Therefore, whether skin potentials are used as an indicator of disease state or non-disease state, it is difficult to achieve high accuracy in using skin potentials to differentiate between bipolar depression and depression because of these factors. Future studies could consider recruiting older depressed patients who have experienced more than two depressive episodes and have previously responded to a single antidepressant to collect SP data and validate the discrimination between MDD and BPD.

The physiological mechanisms underlying skin electrical potential generation remain unclear, and the oxidative stress hypothesis has emerged as one potential explanation. Our study found a significant positive correlation between SP measures and blood prealbumin levels in four tasks among BPD patients. Additionally, SP characteristics in depressed patients were positively correlated with various oxidative stress-related blood indicators, including prealbumin, uric acid, indirect bilirubin, direct bilirubin, and albumin. These findings support the hypothesis that SP abnormalities in individuals are closely related to their oxidative stress status of individuals. The selected indicators in the blood that reflect the level of oxidative stress were based on previous studies in the literature [34–37]. In addition to enzymatic antioxidants such as superoxide dismutase, non-enzymatic antioxidants, including uric acid, bilirubin, and albumin play a crucial role in the body’s endogenous antioxidant defense system. These non-enzymatic antioxidants account for more than 85% of the antioxidant capacity in plasma [36]. Prealbumin, known as transthyretin, which is a carrier protein for the transport of thyroxine and retinol and is also considered a non-enzymatic antioxidant [37]. Oxidative stress, which reflects an imbalance between reactive oxygen species and antioxidant mechanisms, has been suggested to be closely associated with MDD and BD [25]. Bartoli, et al. [38] found that patients with BD had significantly higher levels of uric acid compared to other psychiatric disorders [38]. Another study found that plasma bilirubin was lower in patients with BD compared to healthy controls [39]. Alice et al. found that non-enzymatic antioxidants may have value in differentiating bipolar disorder from depression with 74.9% accuracy based on a decision tree model. And patients with elevated indirect bilirubin and decreased direct bilirubin suggest a higher risk of being diagnosed with BD [40]. The evidence from these previous studies suggests that skin potential merits further investigation as a non-invasive, potentially highly disease-recognizing electrophysiological indicator of affective disorder pathology.

### Limitations

Several limitations should be considered in interpreting the findings of this study. Firstly, we did not collect SP data during the resting state (i.e., non-task) of participants, focusing solely on the task state. Future studies should include the collection of resting state data and compare it with the task state to gain a more comprehensive understanding of SP characteristics. Secondly, due to the exploratory nature of this study, we were unable to strictly match the age of participants across groups. While the impact of age on SP remains uncertain, it would be valuable to analyze the potential effects of covariates such as age and current emotional experience on SP characteristics. Although previous research has not provided conclusive evidence regarding the influence of age on SP, it is worth exploring in future studies. Additionally, while facial videos with eye movements were recorded during the experiment, data on participants’ self-reports of their emotional responses to the presented stimuli were not collected. Therefore, further investigations analyzing the possible effects of covariates, including age and current emotional experience, on SP characteristics are warranted. Thirdly, this study employed a cross-sectional design, and the persistence of SP abnormalities in BPD and MDD patients during the task state as their condition improves remains unknown. Longitudinal studies are needed to investigate the temporal dynamics of SP abnormalities in these patients. Lastly, the present study did not examine the differences in SP characteristics between patients with affective disorders and patients with other psychiatric disorders. Future research should include comparisons with other non-affective psychiatric disorders to further validate the specificity of SP abnormalities in affective disorders.

## Conclusion

Patients with depression and bipolar depression have abnormalities in task-state skin potential that partially reflect the pathological mechanism of the illness, and the abnormalities are potential biological markers of affective disorders.

### Supplementary information

supplementary materials

## Data Availability

The data supporting the results of this study are available on reasonable request from the corresponding author SH.
