# Supplementary material for: Task-state skin potential abnormalities can distinguish major depressive disorder and bipolar depression from healthy controls
Source: Transl Psychiatry. 2024 Feb 23;14:110. doi: 10.1038/s41398-024-02828-9 (PMC10891315; doi:10.1038/s41398-024-02828-9)
Supplement: Supplementary file 1 — supplementary materials [file 41398_2024_2828_MOESM1_ESM.pdf]

Supplementary

Figure S1. Introduction to the acquisition process of skin potential data................ - 2 -

Table S1. Introduction of the 6 stimulation tasks. .... - 3 -

Table S2. Introduction of Cognitive Functioning Assessment .... - 5 -

Table S3. Comparison of characteristics of skin potentials in patients with BPD, MDD, and healthy controls in the free-viewing task. .... - 7 -

Table S4. Comparison of characteristics of skin potentials in patients with BPD, MDD, and healthy controls in the positive and negative emotion recognition task .... - 8 -

Table S5. Comparison of characteristics of skin potentials in patients with BPD, MDD, and healthy controls in the semantic stimulus task.. - 9 -

Table S6. Comparison of characteristics of skin potentials in patients with BPD, MDD, and healthy controls in the situational intervention task................................ - 10 -

Table S7. Comparison of characteristics of skin potentials in patients with BPD, MDD, and healthy controls in the emotional induction task .... - 11 -

Table S8. Comparison of characteristics of skin potentials in patients with BPD, MDD, and healthy controls in the text context stimulation task............................... - 12 -

Table S9. Accuracy of different discriminant models to distinguish among patients with BPD, MDD, and healthy controls based on skin potential characteristics ..... - 13 -

Figure S2. Correlation of skin potential characteristics with blood stress-related indicators in patients with major depressive disorder.................................... - 14 -

Figure S3. Correlation of skin potential characteristics with blood stress-related indicators in patients with bipolar depressive disorder................................. - 15 -

Figure S4. Top 10 variables ranked by feature importance in Support Vector Machine models. .... - 16 -

Figure S1. Introduction to the acquisition process of skin potential data.

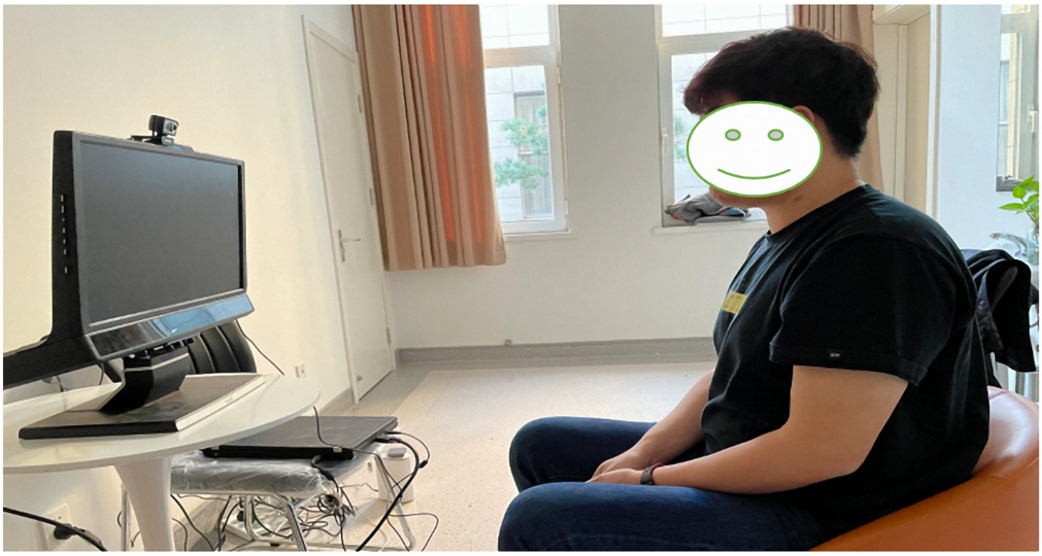

All participants were seated about 1 m from the screen in a quiet and properly lit room. Participants were asked to sit as straight as possible with their hands flat on their legs or on the table. Their feet were placed flat on the floor with their eye level at 1/3 of the height of the screen. The subject's left hand was connected to the portable skin potential collector box in advance and the participant was asked to remain as quiet as possible. The experimental site and the participants' readiness are shown in the figure above.

Table S1. Introduction of the 6 stimulation tasks.

No.	Experimental Task	Stimuli	Procedure
1	Free-viewing task	Twenty neutral pictures without any emotional characteristics selected from GAPED. The pictures include chairs, babies, puppies, flowers, etc.	The participants were freely looking at 20 pictures presented on the screen. Each picture was shown for 10 seconds, for a total of 200 seconds. The free-viewing task aims to observe the emotional state, attention maintenance and skin potential changes when subjects receive external neutral stimuli in a simulated everyday environment.
2	Positive and negative emotion recognition task	Eight pictures with happy expressions and 8 pictures with sad expressions selected from JACFEE	The participants were freely looking at 16 new pictures which composed of random happy-sad pairs presented on the screen. Each picture plays for 3 seconds, for a total of 96 seconds. The task was designed to observe the participants' emotional responses, attentional bias and skin potential changes under simultaneous stimulation of positive and negative emotional expressions.
3	Semantic stimulus task	Eight pictures of happy expressions and 8 pictures of sad expressions selected from the JACFEE gallery that are different from the previous task.	The participant was informed that a number of face-word pictures would be presented on the screen and was asked to look at them freely and judge whether they were right or wrong. They were not allowed to close their eyes or look off-screen during the experiment. Each picture was shown for 3 seconds, for a total duration of 96 seconds. The semantic stimulus paradigm was designed to observe the emotional response, the ability to suppress cognitive interference and the changes in skin potential when the semantic and face stimuli were identical or opposite.
4	Situational intervention task	Four portrait pictures with happy emotions, 4 portrait pictures with sad emotions, 16 pictures of happy scenes, and 16 pictures of sad scenes selected from the free image gallery on the web. Cross-mix and merge the images. The portrait in the foreground of the image carries the same or different emotions as the scene in the background.	Each picture was played for 5 seconds, for a total duration of 240 seconds. The task was designed to observe the subject's emotional response and changes in skin electrical potential during the task when the stimulus pictures carried the same or opposite emotion in the foreground and background.
5	Emotional induction task	Twenty positive images with a validity greater than 6 were selected from the IAPS image library, which included pictures of happy emotions such as food and animals, and exciting pictures such as extreme sports. Twenty negative images with a validity of less than 4.5 were selected, including sad images such	Each video was played for about 60 seconds and each picture was played for 3 seconds, for a total duration of 417 seconds. The task was designed to observe the participants' emotional responses to different emotion-evoking stimuli in the form of videos and pictures, as well as the changes in task-state skin electrical potential.

		as hunger and parting, and bloody images such as fighting and war. In addition, Four movie clips were selected, containing happy emotions, sad emotions, extreme sports-related content, and gore content. One movie clip was matched with 10 images of the same emotion, resulting in 4 types of stimuli.	
6	Text context stimulation task	Ten text fragments describing happy scenes and ten text fragments describing sad scenes from Chinese literature were selected.	The participant was asked to read the text on the monitor aloud, without closing their eyes or looking away. Each text segment was played for 20 seconds, for a total duration of 220 seconds. The aim of the task was to observe the participants' emotional responses to different emotion-evoking stimuli in the form of words and the changes in the electrical skin potential of the task state.

Note:
GAPED: Geneva Affective Picture Database (http://www4.ujaen.es/~erpadial/GAPED.html);
JACFEE: The Japanese and Caucasian Facial Expressions of Emotion (https://www.humintell.com/research-tools/);
IAPS: International Affective Picture System (https://csea.phhp.ufl.edu/media/iapsmessage.html).

Table S2. Introduction of Cognitive Functioning Assessment

(1) Trail Making Test (TMT)
The Connecting the Dots test is a brief timed test that is an indicator of the speed of information processing. The test is divided into two parts: Part A is a practice module, in which 1-8 Arabic numbers are scattered on a piece of paper and the participant is expected to connect all the numbers in order, and Part B is a formal module, in which 1-25 Arabic numbers are scattered on an A4-sized piece of paper and the participant is expected to connect them in order and record the time spent. The test was administered and stopped after 300 seconds, regardless of whether the subject finished.
(2) Symbol Coding Subtest (SCS)
The symbol coding test is likewise one of the indicators to measure the speed of information processing. A sheet of paper is distributed with a digit-symbol correspondence table and a digit-symbol completion table. The test is divided into two parts: Part A is a practice module in which the participant is asked to complete the first 10 spaces in the number-symbol correspondence table according to the rules of the number-symbol correspondence table, and Part B is a formal module in which the participant is asked to fill in as many numbers as possible correctly in the spaces below the corresponding symbols within 90 seconds. The researcher recorded the number of correct answers as the test result.
(3) Hopkins Verbal Learning Test—Revised (HVLT-R)
The Hopkins verbal learning test is an indicator of language learning. An A4-sized sheet of paper contains the words "lion," "emerald," "horse," "tent," "sapphire", "hotel", "cave", "jade", " tiger", "pearl", "cow", "hut", a total of 12 Chinese words. The test was administered as follows: the participant read the 12 words aloud at a speed of 2 seconds per word and was asked to memorize them carefully and recite as many words as possible at the end of the reading. Participants were asked to repeat the words three times within a certain period of time, and the number of correct recitations was recorded at the end of each session and the sum was calculated as the test result.
(4) Neuropsychological Assessment Battery, mazes subtest (MAZES)
Maze tests are often used to measure reasoning and problem-solving skills and have the advantage of being highly practical. A sheet of paper presents a maze pattern with "start" and "end" positions marked. The test is divided into 7 trials, each with 7 progressively more difficult mazes. The test procedure is as follows: the participant is asked to draw a road map with a signature pen from the "start" position, without cutting corners or crossing straight lines to the "end" position, and the time required to complete the maze is recorded. When the test is conducted, the time required to complete the maze is converted into a numerical value according to the maze scoring rule and the total score is calculated.
(5) The Stroop Color and Word Test (SCWT)
The Stroop Color Word Test is a widely used neuropsychological test that is commonly used to assess the ability to suppress cognitive interference. The test is divided into three test sections: a word test, a color test, and a color word test. The specific process of the test was as follows: each part was divided into a practice module and a formal module. In the word test, subjects were asked to read the Chinese characters as accurately and quickly as possible, and the time taken was recorded. For the color test, participants were asked to read the color as accurately and quickly as possible, and the time taken was recorded. For the color word test, participants were asked to read the corresponding color as accurately and quickly as possible without interference

from the meaning of the word, and the time taken was recorded.
(6) Continuous Performance Test-Identical Pair (CPT-IP)
The continuous performance test is a computerized test that is commonly used to measure maintenance, focused attention, or alertness. The test is divided into practice trials, two-digit trials, three-digit trials, and four-digit trials with increasing difficulty. The test is administered as follows: when a sequence of random numbers is presented on a computer monitor for a short period of time, the participant memorizes and evaluates them and is asked to press the left button once when the currently displayed number is the same as the previous number. The computer software automatically records the subject's performance and converts it into numbers that can be used for comparison across subjects.

Table S3. Comparison of characteristics of skin potentials in patients with BPD, MDD, and healthy controls in the free-viewing task.

	BPD (n=77)	MDD(n=53)	HC（n=79）	F	*P*	Corrected *P* value BPDvsHC	MDDvsHC	BPDvsMDD
task1.max	-6.059±21.609	-8.885±21.761	-6.797±19.668	0.296	0.744			
task1.min	-30.323±19.548	-33.467±22.543	-28.933±19.684	0.795	0.453			
task1.n50	0.427±0.156	0.435±0.145	0.454±0.166	0.624	0.537			
task1.mean	0.447±0.113	0.443±0.107	0.464±0.113	0.736	0.480			
task1.var	0.059±0.025	0.057±0.024	0.061±0.025	0.447	0.640			
task1.rms	0.510±0.109	0.503±0.107	0.526±0.112	0.792	0.454			
task1.diff1_mean	$1.223*10^{-4}\pm7.560*10^{-4}$	$2.459*10^{-4}\pm7.225*10^{-4}$	$1.006*10^{-4}\pm6.760*10^{-4}$	0.723	0.487			
task1.diff2_mean	$-1.366*10^{-6}\pm4.650*10^{-5}$	$-1.101*10^{-5}\pm6.472*10^{-5}$	$-2.413*10^{-6}\pm3.703*10^{-5}$	0.701	0.497			
task1.diff1_std	0.020±0.010	0.021±0.011	0.017±0.008	2.763	0.065			
task1.diff2_std	0.021±0.015	0.019±0.014	-0.015±0.010	3.947	0.021*	0.018*	0.189	0.905
task1.freq0	2.559±0.563	2.459±0.534	2.632±0.598	1.464	0.234			
task1.freq1	0.134±0.082	0.154±0.094	0.131±0.067	1.096	0.336			
task1.freq2	0.081±0.057	0.105±0.075	0.074±0.043	4.408	0.013*	0.854	0.034*	0.158
task1.freq3	0.061±0.042	0.073±0.051	0.062±0.038	1.403	0.248			
task1.freq4	0.049±0.037	0.065±0.045	0.047±0.031	4.093	0.018*	0.985	0.046*	0.105
task1.freq5	0.031±0.023	0.042±0.032	0.030±0.019	4.126	0.017*	0.976	0.059	0.131
task1.freq6	0.022±0.015	0.027±0.019	0.022±0.014	2.458	0.088			
task1.freq7	0.021±0.014	0.025±0.017	0.019±0.012	2.185	0.115			

Note. Please see Table 2 for the definition of the skin potential measures. BPD, bipolar depression; MDD, major depressive disorder; HC, healthy control；F：One-way ANOVA；*: P<0.05.

Table S4. Comparison of characteristics of skin potentials in patients with BPD, MDD, and healthy controls in the positive and negative emotion recognition task

	BPD (n=77)	MDD(n=53)	HC（n=79）	F	*P*	Corrected P value		
						BPDvsHC	MDDvsHC	BPDvsMDD
task2.max	-7.484±20.948	-10.079±21.854	-8.897±18.158	0.267	0.766			
task2.min	-27.632±21.280	-29.846±23.482	-27.729±19.484	0.206	0.814			
task2.n50	0.450±0.169	0.458±0.146	0.422±0.158	0.991	0.373			
task2.mean	0.461±0.120	0.473±0.102	0.440±0.115	1.431	0.241			
task2.var	0.060±0.022	0.059±0.022	0.068±0.0262	3.118	0.046*	0.100	0.111	1.000
task2.rms	0.522±0.115	0.533±0.097	0.512±0.113	0.595	0.553			
task2.diff1_mean	$1.890*10^{-4}\pm1.096*10^{-3}$	$1.117*10^{-4}\pm1.118*10^{-3}$	$3.528*10^{-5}\pm1.156*10^{-3}$	0.364	0.695			
task2.diff2_mean	$-4.101*10^{-5}\pm1.197*10^{-4}$	$3.906*10^{-5}\pm1.563*10^{-4}$	$5.481*10^{-6}\pm1.181*10^{-4}$	1.843	0.161			
task2.diff1_std	0.031±0.025	0.033±0.030	0.026±0.025	1.129	0.325			
task2.diff2_std	0.037±0.043	0.037±0.051	0.029±0.045	0.795	0.453			
task2.freq0	2.666±0.707	2.577±0.608	2.625±0.645	0.286	0.752			
task2.freq1	0.161±0.092	0.168±0.086	0.137±0.071	2.741	0.067			
task2.freq2	0.095±0.067	0.112±0.0682	0.087±0.050	2.783	0.064			
task2.freq3	0.080±0.047	0.097±0.054	0.071±0.041	5.114	0.007**	0.624	0.005**	0.127
task2.freq4	0.066±0.041	0.081±0.048	0.059±0.039	4.137	0.017*	0.917	0.014*	0.166
task2.freq5	0.046±0.032	0.052±0.043	0.038±0.024	3.076	0.048*	0.395	0.047*	0.868
task2.freq6	0.033±0.021	0.037±0.026	0.028-02±0.018	2.755	0.066			
task2.freq7	0.030±0.019	0.036±0.021	0.027±0.020	2.828	0.061			

Note. Please see Table 2 for the definition of the skin potential measures. BPD, bipolar depression; MDD, major depressive disorder; HC, healthy control；F：One-way ANOVA；*: P<0.05; ** P<0.01.

Table S5. Comparison of characteristics of skin potentials in patients with BPD, MDD, and healthy controls in the semantic stimulus task

	BPD (n=77)	MDD(n=53)	HC（n=79）	F	*P*	Corrected P value		
						BPDvsHC	MDDvsHC	BPDvsMDD
task3.max	2.466±21.482	-1.307±23.654	2.445±19.600	0.613	0.543			
task3.min	-22.873±21.564	-24.823±24.999	-24.464±20.523	0.154	0.857			
task3.n50	0.464±0.152	0.448±0.152	0.458±0.163	0.172	0.842			
task3.mean	0.472±0.105	0.453±0.111	0.464±0.116	0.452	0.637			
task3.var	0.059±0.024	0.052±0.018	0.055±0.022	1.532	0.219			
task3.rms	0.532±0.097	0.508±0.106	0.522±0.105	0.852	0.428			
task3.diff1_mean	$2.924*10^{-4}\pm1.083*10^{-3}$	$1.728*10^{-4}\pm1.127*10^{-3}$	$3.118*10^{-4}\pm1.138*10^{-3}$	0.272	0.762			
task3.diff2_mean	$-1.422*10^{-5}\pm1.237*10^{-4}$	$-1.772*10^{-5}\pm1.381*10^{-4}$	$3.025*10^{-5}\pm1.146*10^{-4}$	0.418	0.659			
task3.diff1_std	0.029±0.012	0.035±0.0171	0.028±0.011	4.398	0.013*	0.965	0.047*	0.111
task3.diff2_std	0.025±0.015	0.033±0.030	0.021±0.013	6.534	0.002**	0.248	0.019*	0.169
task3.freq0	2.421±0.611	2.492±0.604	2.512±0.497	0.533	0.588			
task3.freq1	0.186±0.103	0.207±0.071	0.203±0.074	1.192	0.306			
task3.freq2	0.141±0.086	0.146±0.072	0.145±0.066	0.075	0.928			
task3.freq3	0.111±0.077	0.128±0.063	0.112±0.050	1.232	0.294			
task3.freq4	0.107±0.073	0.116±0.065	0.118±0.064	0.573	0.565			
task3.freq5	0.063±0.041	0.062±0.030	0.064±0.030	0.044	0.957			
task3.freq6	0.040±0.024	0.044±0.024	0.038±0.018	1.203	0.302			
task3.freq7	0.036±0.025	0.044±0.023	0.034±0.016	3.326	0.038*	0.890	0.030*	0.224

Note. Please see Table 2 for the definition of the skin potential measures. BPD, bipolar depression; MDD, major depressive disorder; HC, healthy control；F：One-way ANOVA；*: P<0.05.

Table S6. Comparison of characteristics of skin potentials in patients with BPD, MDD, and healthy controls in the situational intervention task

	BPD (n=77)	MDD(n=53)	HC（n=79）	F	*P*	Corrected P value BPDvsHC	MDDvsHC	BPDvsMDD
task4.max	-3.170±19.497	-6.802±22.025	-4.391±17.990	0.542	0.582			
task4.min	-23.396±18.008	-26.020±22.089	-23.283±19.756	0.365	0.695			
task4.n50	0.452±0.161	0.460±0.142	0.471±0.172	0.258	0.773			
task4.mean	0.467±0.111	0.469±0.102	0.472±0.128	0.048	0.953			
task4.var	0.061±0.024	0.059±0.021	0.057±0.023	0.661	0.518			
task4.rms	0.529±0.107	0.529±0.094	0.530±0.125	0.002	0.998			
task4.diff1_mean	$-2.025*10^{-4}\pm1.156*10^{-3}$	$-4.432*10^{-4}\pm1.046*10^{-3}$	$-2.191*10^{-4}\pm1.059*10^{-3}$	0.898	0.409			
task4.diff2_mean	$-5.577*10^{-6}\pm8.068*10^{-5}$	$-2.185*10^{-5}\pm9.289*10^{-5}$	$1.559*10^{-5}\pm6.055*10^{-5}$	3.879	0.022*	0.266	0.021*	0.718
task4.diff1_std	0.025±0.014	0.027±0.014	0.021±0.015	2.604	0.076			
task4.diff2_std	0.028±0.023	0.029±0.023	0.023±0.025	1.396	0.250			
task4.freq0	2.797±0.583	2.828±0.590	2.821±0.595	0.052	0.950			
task4.freq1	0.149±0.087	0.158±0.085	0.123±0.081	3.144	0.045*	0.182	0.064	1.000
task4.freq2	0.093±0.069	0.107±0.064	0.081±0.057	2.610	0.076			
task4.freq3	0.074±0.050	0.086±0.054	0.063±0.044	3.343	0.037*	0.533	0.032*	0.537
task4.freq4	0.063±0.044	0.071±0.044	0.053±0.040	3.006	0.052			
task4.freq5	0.039±0.027	0.047±0.032	0.035±0.026	2.955	0.054			
task4.freq6	0.030±0.020	0.032±0.018	0.026±0.020	1.475	0.321			
task4.freq7	0.027±0.017	0.029±0.017	0.024±0.018	1.321	0.269			

Note. Please see Table 2 for the definition of the skin potential measures. BPD, bipolar depression; MDD, major depressive disorder; HC, healthy control；F：One-way ANOVA；*: P<0.05.

Table S7. Comparison of characteristics of skin potentials in patients with BPD, MDD, and healthy controls in the emotional induction task

	BPD (n=77)	MDD(n=53)	HC（n=79）	F	*P*	Corrected P value		
						BPDvsHC	MDDvsHC	BPDvsMDD
task5.max	0.987±18.081	-3.500±24.146	-4.868±19.394	1.741	0.178			
task5.min	-30.634±19.783	-32.750±23.222	-30.784±19.486	0.195	0.823			
task5.n50	0.435±0.150	0.418±0.130	0.486±0.140	4.292	0.015*	0.078	0.023*	1.000
task5.mean	0.447±0.115	0.429±0.103	0.485±0.106	4.691	0.010**	0.095	0.012*	1.000
task5.var	0.048±0.018	0.044±0.015	0.046±0.018	0.860	0.424			
task5.rms	0.499±0.108	0.477±0.102	0.530±0.107	4.181	0.017*	0.208	0.016*	0.734
task5.diff1_mean	$5.915*10^{-5}±2.856*10^{-4}$	$1.318*10^{-5}±2.188*10^{-4}$	$-1.681*10^{-5}±2.664*10^{-4}$	1.646	0.195			
task5.diff2_mean	$3.073*10^{-6}±1.919*10^{-5}$	$2.729*10^{-6}±2.726*10^{-5}$	$4.893*10^{-7}±1.929*10^{-5}$	0.320	0.727			
task5.diff1_std	0.019±0.010	0.022±0.013	0.018±0.008	2.788	0.064			
task5.diff2_std	0.020±0.017	0.023±0.023	0.016±0.011	2.684	0.071			
task5.freq0	2.513±0.616	2.450±0.560	2.773±0.618	5.660	0.004**	0.023*	0.009**	1.000
task5.freq1	0.134±0.077	0.140±0.067	0.132±0.056	0.227	0.797			
task5.freq2	0.081±0.051	0.091±0.044	0.078±0.037	1.423	0.243			
task5.freq3	0.056±0.033	0.062±0.033	0.056±0.02*	0.712	0.492			
task5.freq4	0.047±0.027	0.051±0.028	0.046±0.024	0.690	0.503			
task5.freq5	0.033±0.020	0.037±0.019	0.032±0.018	1.196	0.305			
task5.freq6	0.024±0.013	0.027±0.014	0.023±0.014	1.347	0.262			
task5.freq7	0.019±0.010	0.022±0.013	0.019±0.011	1.700	0.185			

Note. Please see Table 2 for the definition of the skin potential measures. BPD, bipolar depression; MDD, major depressive disorder; HC, healthy control；F：One-way ANOVA； *: P<0.05; ** P<0.01.

Table S8. Comparison of characteristics of skin potentials in patients with BPD, MDD, and healthy controls in the text context stimulation task

	BPD (n=77)	MDD(n=53)	HC（n=79）	F	*P*	Corrected P value BPDvsHC	MDDvsHC	BPDvsMDD
task6.max	3.840±17.024	0.385±22.968	-2.048±18.325	1.853	0.159			
task6.min	-22.405±18.344	-25.009±25.007	-25.762±19.714	0.550	0.578			
task6.n50	0.448±0.137	0.440±0.154	0.417±0.152	0.906	0.406			
task6.mean	0.455±0.106	0.449±0.119	0.434±0.119	0.665	0.515			
task6.var	0.047±0.019	0.041±0.017	0.042±0.019	2.157	0.118			
task6.rms	0.505±0.101	0.494±0.113	0.481±0.114	0.946	0.390			
task6.diff1_mean	$9.700*10^{-6}$±$4.434*10^{-4}$	$-5.304*10^{-5}$±$5.028*10^{-4}$	$-3.898*10^{-5}$±$4.359*10^{-4}$	0.360	0.698			
task6.diff2_mean	$-1.341*10^{-6}$±$4.434*10^{-5}$	$-1.253*10^{-5}$±$5.531*10^{-5}$	$-7.795*10^{-6}$±$4.950*10^{-5}$	0.846	0.431			
task6.diff1_std	0.025±0.011	0.026±0.012	0.023±0.011	1.441	0.239			
task6.diff2_std	0.023±0.016	0.026±0.021	0.021±0.019	1.365	0.258			
task6.freq0	2.496±0.507	2.505±0.554	2.426±0.535	0.475	0.623			
task6.freq1	0.180±0.091	0.183±0.080	0.17±0.063	0.916	0.402			
task6.freq2	0.124±0.073	0.126±0.062	0.109±0.043	1.680	0.189			
task6.freq3	0.093±0.055	0.095±0.051	0.086±0.039	0.606	0.547			
task6.freq4	0.076±0.046	0.078±0.045	0.069±0.032	0.852	0.428			
task6.freq5	0.047±0.026	0.051±0.028	0.044±0.020	1.206	0.302			
task6.freq6	0.032±0.019	0.035±0.018	0.032±0.015	0.818	0.443			
task6.freq7	0.030±0.019	0.032±0.016	0.029±0.014	0.681	0.507			

Note. Please see Table 2 for the definition of the skin potential measures. BPD, bipolar depression; MDD, major depressive disorder; HC, healthy control；F：One-way ANOVA.

Table S9. Accuracy of different discriminant models to distinguish among patients with BPD, MDD, and healthy controls based on skin potential characteristics

	Accuracy (MDD vs BPD vs HC)	Accuracy (MDD vs HC)	Accuracy (BPD vs HC)	Accuracy (MDD vs BPD)
KNN	0.53	0.78	0.58	0.59
LDA	0.49	0.45	0.52	0.64
**SVM**	**0.59**	**0.78**	**0.65**	**0.69**
LR	0.59	0.69	0.69	0.64
GBDT	0.57	0.72	0.63	0.60

Note: The performance of a discriminant model is measured by sensitivity, specificity, accuracy, area under the ROC curve, etc. The table presents only the accuracy of the model to differentiate between two or three groups for visual comparison. BPD, Bipolar Depression; MDD, major depressive disorder; HC, healthy control; KNN, k-Nearest Neighbor；LDA, Linear Discriminant Analysis；SVM, Support Vector Machine；LR, logistic regression；GBDT, Gradient Boosting Decision Tree.

Figure S2. Correlation of skin potential characteristics with blood stress-related indicators in patients with major depressive disorder.

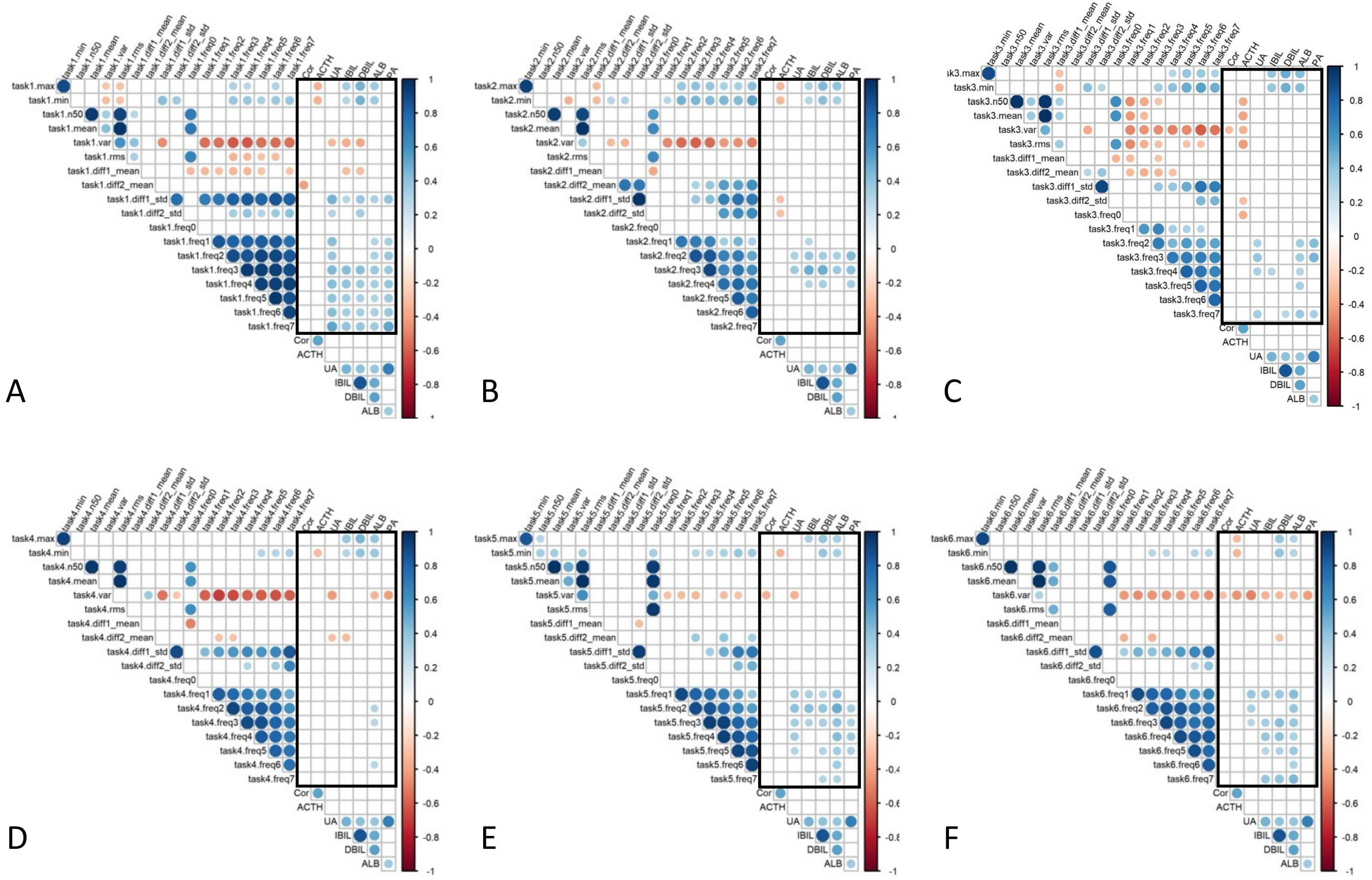

Correlation analysis was performed using the Pearson correlation test. The color band on the right side marks the correlation coefficient r. The colored dots in the boxes where the two variables intersect suggest a significant correlation between them ($P < 0.05$). A larger dot area represents a smaller corresponding p-value. The darker the color of the dots represents the corresponding correlation coefficient closer to 1 or -1. If the box at the intersection is blank, no statistically significant correlation was found between the two variables. Please see Table S1 for task information. Please see Table 2 for the definition of the skin potential measures. Cor, cortisol levels; ACTH, adrenocorticotropic hormone; UA, uric acid; IBIL, indirect bilirubin levels; DBIL, direct bilirubin levels; PA, prealbumin. Figures are numbered A-F, corresponding to stimulation Tasks 1-6, respectively.

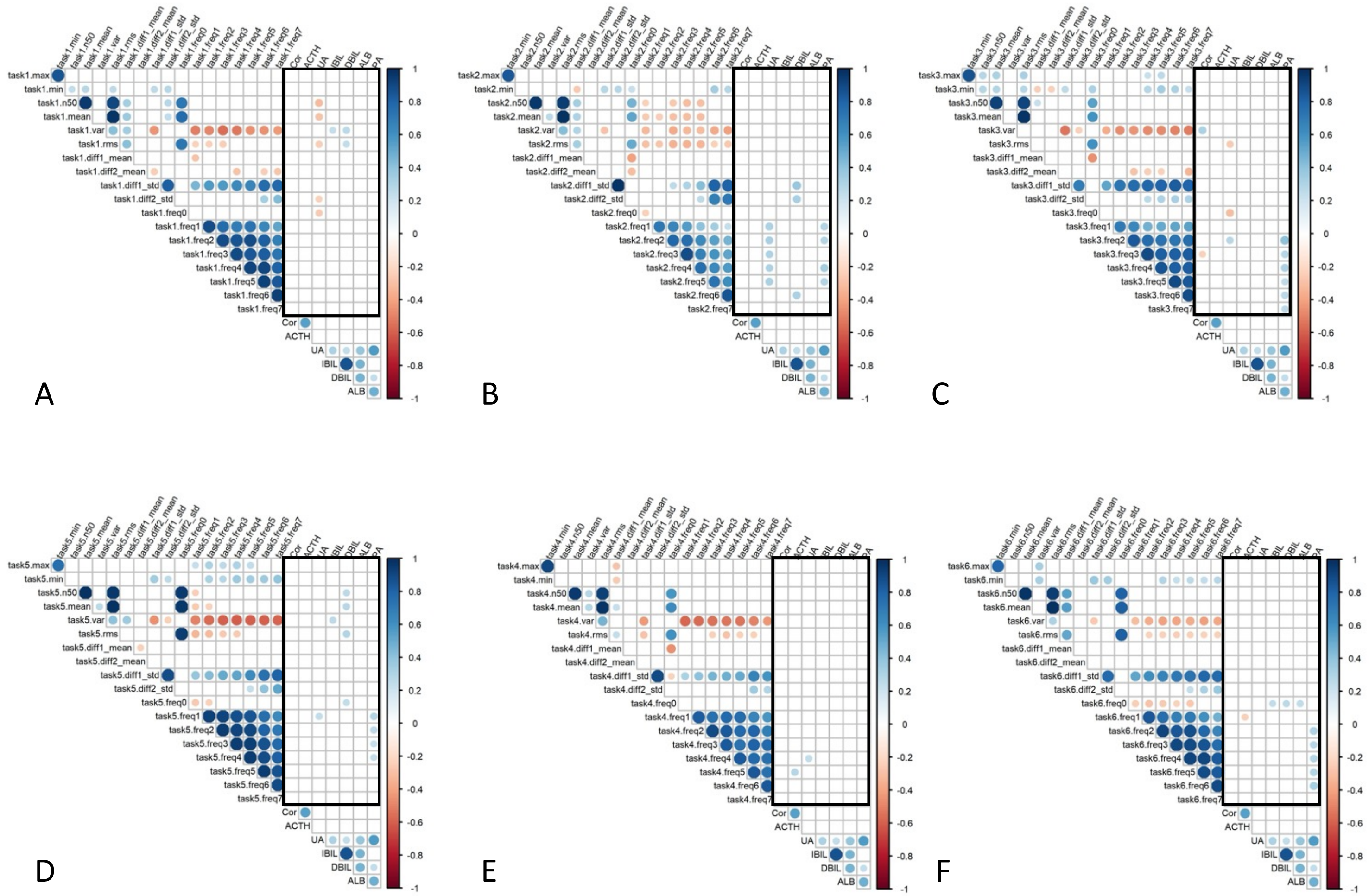

Figure S3. Correlation of skin potential characteristics with blood stress-related indicators in patients with bipolar depressive disorder.Correlation analysis was performed using the pearson correlation test. The color band on the right side marks the correlation coefficient r. The colored dots in the boxes where the two variables intersect suggest a significant correlation between them (*P* < 0.05). A larger dot area represents a smaller corresponding p-value. The darker the color of the dots represents the corresponding correlation coefficient closer to 1 or -1. If the box at the intersection is blank, no statistically significant correlation was found between the two variables. Please see Table S1 for task information. Please see Table 2 for the definition of the skin potential measures. Cor, cortisol levels; ACTH, adrenocorticotropic hormone; UA, uric acid; IBIL, indirect bilirubin levels; DBIL, direct bilirubin levels; PA, prealbumin. Figures are numbered A-F, corresponding to stimulation Tasks 1-6, respectively.

Figure S4. Top 10 variables ranked by feature importance in Support Vector Machine models.

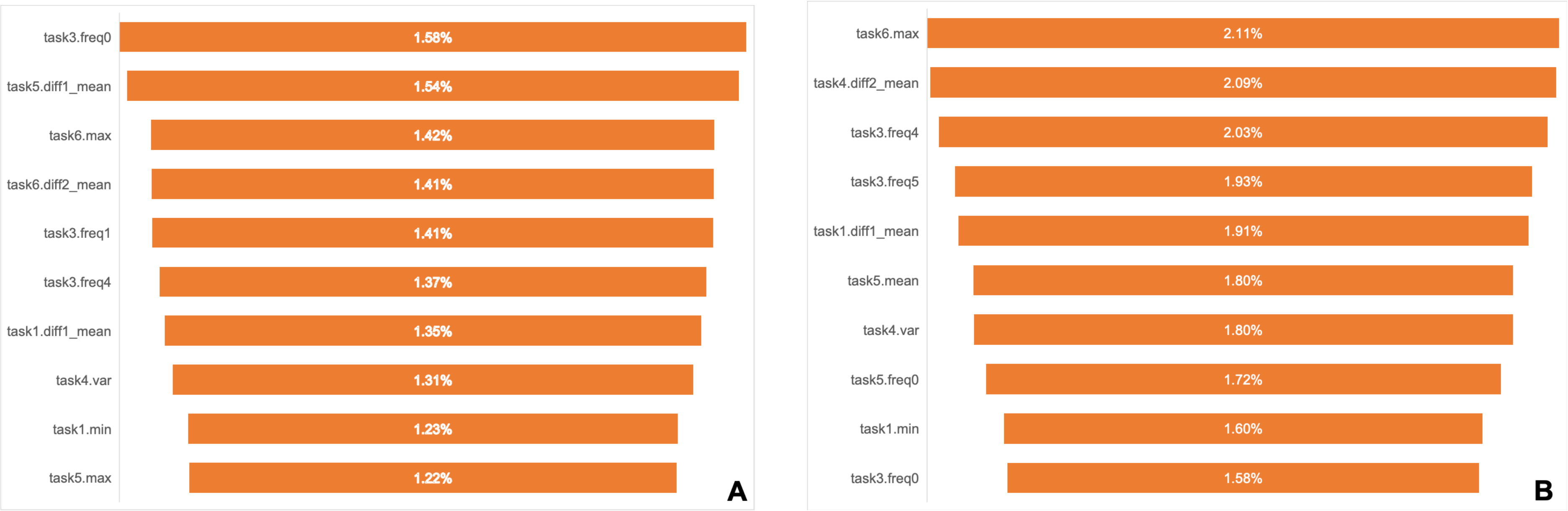

Figure A shows the top 10 skin potential variables ranked by feature importance in the SVM model used to differentiate between bipolar depression, MDD, and healthy controls. Figure B shows the top 10 skin potential variables ranked by feature importance in the SVM model used to distinguish between MDD and healthy controls. The Permutation Importance method is utilized to evaluate the significance of features in Support Vector Machine (SVM) models. This method calculates feature importance by randomly permuting feature values and measuring the resulting change in model performance after training the SVM model. The process involves the following steps: (1) Predicting on the original data using the SVM model and recording the baseline performance metric, such as accuracy. (2) The values of each feature are randomly shuffled or permuted to create a new feature permutation. (3) Predictions are made using the permuted feature set, and the performance metric is calculated. (4) The feature importance score is computed as the difference between the baseline performance and the permuted performance. Larger differences indicate greater contributions of the feature to the model's performance. (5) The process is repeated several times to obtain more stable and reliable estimates of feature importance. The Permutation Importance method is based on the concept that important features have a significant impact on model predictions. This method quantifies the contribution of each feature to the model by disrupting the true relationship between the feature and the target through random permutations.
